# 

**Published:** 1914-02-28

**Authors:** 


### CONTENTS.

PAGE

**British Hospital Progress—1887-1913** ... ... 577

**Poor-Law Guardians and the Feeble-Minded** 578

**Hospital and Institutional News** ... ... ... 579
Salvarsan Wards in Australia — Interruption at an Annual Meeting—"No Fighting in the Board Room"—A Defence from North Evington Infirmary—Sanatoria Beds for Insured Patients—The Scandal of Domiciliary Benefit—Mockery by an Insurance Committee—The Cameron Prize for Professor Ehrlich—A Chairman as Hospital Historian—Sanatoria and Convalescent Homes—The Craze for Spoon-fed Legislation—A Great Opportunity—The Ansell Reversion—A Case for Inquiry at Camberwell—A Notable Chairman's Resignation—An Experiment in Medical Relief—The County Authorities and Worcester Infirmary—The Penalty of a Despairing Policy—The Trained Nurses' Annuity Fund—Proposals it is Wise to Abandon—Retirement of a Hospital Secretary—A Sudden Increase in Hospital Expenses—Dover Coal—New Chairman of the Saturday Fund—A Hospital Secretary's Savings—Closing of Halifax Eye Hospital—This Week's Drug Market.

**Modern Methods Series** ... ... ... ... 585

The Surgery of Malignant Disease.

**Appointments** ... ... ... ... ... ... 585

**The Liverpool Psycho-Therapeutic Institution** 586

By SIDNEY WILKINSON, M.R.C.S., L.R.C.P.

**The Campaign Against Syphilis** ... ... ... 587

An Anticipation of the Royal Commission's Report.

**Research and Remedies** ... ... ... ... 588

### CONTENTS—(*continued*).

PAGE

**Reports on Hospitals of the United Kingdom—Series III.** ... ... ... ... ... ... 589
By SIR HENRY BURDETT, K.C.B., K.C.V.O
The Royal Surrey County Hospital, Guildford.

**Buildings Contemplated** ... ... ... ... ... 590

**National Insurance Act Developments** ... ... 591
Benefits for Exempt Persons.

**Workpeople's Contributions to Hospitals** ... 593
Threatened with Legislative Destruction.

**Gifts and Bequests** ... ... ... ... ... ... 594

**Round the World** ... ... ... ... ... ... 595

Fellow-Workers and their Doings.

**The Origin and Evolution of the 18th Century Hospital Movement** ... ... ... ... ... 596

**Another Light on the Milk Problem** ... ... 597
The Keeping Properties of Condensed Milks.

**The Central Poor-Law Conference** ... ... 598
Boards of Guardians and the Insurance Act.

PAGE

**The Editor's Letter-Box** ... ... ... ... ... 599

Treatment of Sciatica—Race Improvement.

**The Mental Hospital Service** ... ... ... 600

Action by the Medical Officers.

**Institutional Needs** ... ... ... ... ... ... 600

Polylactol.

**Automatic Telephones for Institutions** ... 601
The Composite Installation at New "King's."

**The National Medical Union (Official)** ... ... 602
The Profession of Medicine.

**The Institutional Worker** ... ... (Suppl.) 1

Designs for Coal Bunkers.

Comparative Values of Meat.

**Our Bureau of Information** ... ... ,, 2
Questions for February.
Hospital Worthies.
The Sick and in Need.
Employment and Training
Approved Homes.

